# Adverse Childhood Experiences among American Indian/Alaska Native Children: The 2011-2012 National Survey of Children's Health

**DOI:** 10.1155/2016/7424239

**Published:** 2016-07-26

**Authors:** Mary Kay Kenney, Gopal K. Singh

**Affiliations:** U.S. Department of Health and Human Services, Health Resources and Services Administration, Maternal and Child Health Bureau, Rockville, MD 20857, USA

## Abstract

We examined parent-reported adverse childhood experiences (ACEs) and associated outcomes among American Indian and Alaska Native (AI/AN) children aged 0–17 years from the 2011-2012 National Survey of Children's Health. Bivariate and multivariable analyses of cross-sectional data on 1,453 AI/AN children and 61,381 non-Hispanic White (NHW) children assessed race-based differences in ACEs prevalence and differences in provider-diagnosed chronic emotional and developmental conditions, health characteristics, reported child behaviors, and health services received as a function of having multiple ACEs. AI/AN children were more likely to have experienced 2+ ACEs (40.3% versus 21%), 3+ ACEs (26.8% versus 11.5%), 4+ ACEs (16.8% versus 6.2%), and 5+ ACEs (9.9% versus 3.3%) compared to NHW children. Prevalence rates for depression, anxiety, and ADHD were higher among AI/AN children with 3+ ACEs (14.4%, 7.7%, and 12.5%) compared to AI/ANs with fewer than 2 ACEs (0.4%, 1.8%, and 5.5%). School problems, grade failures, and need for medication and counseling were 2-3 times higher among AI/ANs with 3+ ACEs versus the same comparison group. Adjusted odds ratio for emotional, developmental, and behavioral difficulties among AI/AN children with 2+ ACEs was 10.3 (95% CI = 3.6–29.3). Race-based differences were largely accounted for by social and economic-related factors.

## 1. Introduction

A variety of deleterious child health and well-being outcomes have connections with adverse or traumatic experiences in childhood. Multiple experiences of food insufficiency and hunger are associated with behavioral, emotional, and academic problems and children exposed to family substance abuse and domestic violence show higher levels of aggression, delinquency, hyperactivity, impulsivity, anxiety, negative affectivity, and posttraumatic stress disorder compared to children without such histories [1–5]. Children exposed to maltreatment, family dysfunction, or caregiver loss frequently meet criteria for attention deficit hyperactivity disorder (ADHD) as well as conduct, anxiety, communication, and reactive attachment disorders [6]. A connection between levels of adversity in childhood and comorbid physical, mental, and substance abuse disorders in adulthood suggested that cumulative disadvantage is predictive of cumulative dysfunction [7, 8].

It has been stated that American Indians and Alaska Natives (AI/ANs) are disproportionally affected by childhood trauma, including abuse, neglect, and family violence, with pronounced disparities between White and AI/AN youth, sometimes attributed to cultural degradation resulting from multigenerational historical colonization and trauma [9]. One study of Native American adolescents and young adults from the Northern Plains states indicated that approximately half of the sample had been exposed to one or more severe traumatic events [10]. However, many AI/AN studies of outcomes for traumatized children have been conducted with nonrepresentative samples of adults or adolescents reporting on past experiences and examining narrowly defined health outcomes [10–13]. None have examined difficulties across a range of developmental, emotional, and behavioral problems experienced by younger children of varying ages.

The purpose of this study was to examine the prevalence of parent-reported adverse childhood experiences (ACEs) in a population-based nationally representative sample of AI/AN children from the 2011-2012 National Survey of Children's Health (NSCH). We examine ACEs in AI/AN children across the entire pediatric age spectrum of 0–17 years and report on the associated emotional, developmental, and behavioral outcomes. Based on previous literature, it is hypothesized that (1) AI/AN race will be associated with greater accumulation of ACEs compared to a reference population of non-Hispanic White (NHW) children and (2) the increased accumulation of ACEs will be associated with an increasing gradient of parent-reported health problems and need for services among AI/AN children 0–17 years of age. Due to the high rates of mortality/morbidity among AI/ANs, including PTSD, suicide, and vehicular or violent injuries and death in adolescents and young adults, it is essential to find their roots in childhood to better prevent a self-perpetuating cycle of physical and behavioral health problems.

## 2. Methods

### 2.1. Population and Data

The NSCH is a quadrennial random-digit-dialing household survey that was designed to produce national and state-specific prevalence estimates of an array of variables concerning children and family (parental) health; children's physical, emotional, and behavioral development; family stress and coping behaviors; family activities; and parental concerns about their children. Of the original 95,677 cases in the survey, exclusions were made for cases with missing data for the adverse childhood experiences outcome measure, resulting in a sample of 94,520. The subpopulations of inference for the current analyses were 1,453 AI/AN 0–17-year-olds and a comparison group of 61,381 NHW children. The remaining sample was comprised of children of other races/ethnicities and was not included in this analysis. No oversampling of minority populations was conducted. Child-level household surveys were conducted with parents or guardians under the leadership of the Maternal and Child Health Bureau, Health Resources and Services Administration (HRSA), U.S. Department of Health and Human Services, and implemented through the National Center for Health Statistics, Centers for Disease Control and Prevention (CDC), U.S. Department of Health and Human Services.

All survey items/questions on the NSCH were developed under the direction of two technical expert working groups. The items were finalized after repeated rounds of cognitive testing as well as best practice language translation and pilot testing through the National Center for Health Statistics. All survey items used in this study have been documented previously and their properties are presented in publicly available manuals [14]. Data were weighted to represent the population of noninstitutionalized children aged 0–17 nationally and in each state. The National Center for Health Statistics Research Ethics Review Board approved all data collection procedures for the survey.

### 2.2. Key Measures

The independent variables of interest included 9 different parent-reported ACEs calculated as individual rates and as accumulated rates from responses to the following survey questions: Has [child's name] ever (1) lived in a household with difficulty affording food or housing, (2) lived with a parent that had gotten divorced/separated, (3) lived with a parent who died, (4) lived with a parent who served time in jail, (5) seen parents hit, kick, slap, punch, or beat each other up, (6) been a victim of violence/witness to violence in [his/her] neighborhood, (7) lived with anyone who was mentally ill, suicidal, or severely depressed for more than a couple of weeks, (8) lived with anyone who had a problem with alcohol/drugs, and (9) been treated/judged unfairly based on race/ethnicity? The 9 NSCH adverse childhood experience survey items were based on items in the seminal work on adverse childhood experiences, with modifications overseen by a technical expert panel and evaluated through standard survey item testing through the National Center for Health Statistics [8]. Response alternatives were “yes/no” for most of the items with the exception of economic hardship and racial/ethnic discrimination experiences. Responses of “somewhat often” or “very often” (in contrast to “rarely” or “never”) were coded as ever experiencing economic hardship and racial/ethnic discrimination.

Other key measures included the health, developmental, and service outcomes having possible associations with multiple ACEs. The survey questions and response alternatives on which these variables were based are listed in Table 1. These included (1) provider-diagnosed disorders (i.e., learning disability, depression, anxiety disorder, conduct disorder, autism spectrum disorder, attention deficit/hyperactivity disorder, developmental delay, and speech disorder) reported by the parent; (2) other parent-reported health characteristics (i.e., overall rate of special health care needs, prescription medication usage, elevated service use, functional limitations, special therapy usage, and emotional, developmental, or behavioral disorders requiring mental health treatment); (3) parent-reported behaviors in the 0–5-year-old population (i.e., acquiring independence, learning in preschool, and behaving/getting along with others); (4) parent-reported behaviors in the 6–17-year-old population (i.e., school problems, frequent arguing, acting cruel/mean to others, unhappy, sad, or depressed affect, lack of control, investment in schoolwork, and grade repetition); and (5) health/services received (i.e., insured adequately, received needed counseling, screened by a doctor for developmental problems, screened for parent-reported developmental concerns, and received an Individualized Family Services Plan (IFSP) or Individualized Education Program (IEP)).

Finally, an additional outcome measure for logistic regression analysis was the proportion of parent-reported emotional, developmental, or behavioral difficulties. This was derived from one of the previously mentioned 5 questions used in a screening battery for children with special health care needs (i.e., Does [child's name] have any kind of emotional, developmental, or behavioral problem for which [he/she] needs treatment or counseling? Has [his/her] emotional, developmental, or behavioral problem lasted or is it expected to last 12 months or longer?). Response alternatives were “yes/no.”

### 2.3. Sample Demographic Characteristics

Sociodemographic characteristics of the sample (Table 2) selected for race-group comparison included birth weight (very low or <1500 grams, low or 1500–2500 grams, and not low); gestational age (within normal limits, ≥3 weeks premature); gender (male, female); age (0–5, 6–11, and 12–17 years); status of child with/without special health care needs which is a dichotomous variable based on a positive response to at least one of 5 survey items (i.e., child uses prescription medicine, needs special therapy, has elevated service use, has ongoing emotional, developmental, or behavioral conditions, or has limitations on activity); poverty status (<100%, 100%–199%, 200%–399%, and ≥400% of the Federal Poverty level based on the U.S. Department of Health and Human Services Federal Poverty Guidelines for 2012); family structure (two parents, biological; two parents, step family; single mother, no father present; other); mother's age (≤30 years, 31–45 years, or >45 years); highest household educational attainment (<high school, high school, and >high school); insurance coverage type (public, private, or none); medical home which is a dichotomous composite based on five component variables (i.e., having a personal doctor or nurse, having a usual source for sick and well care, having family-centered care, having no problems getting needed referrals, and receiving effective care coordination when needed) developed from 19 separate questions; neighborhood support, a dichotomous composite variable based on 4 survey items (i.e., people in the neighborhood help each other out, watch out for each other's children, can be counted on, and can be trusted to help a neighbor child who was outside playing and got hurt or scared); metropolitan status (metropolitan/nonmetropolitan residence); and AI/AN region (Alaska, East, Northern Plains, Pacific Coast, or Southwest). Weighted frequencies and prevalence were determined for the above characteristics for the AI/AN and NHW subpopulations.

### 2.4. Data Analysis Plan

We conducted the analyses of AI/AN ACEs and their relationships with a variety of demographic, health, developmental, and service factors in several steps. First, we determined differences between AI/AN children and a reference population (NHW children) in individual and accumulated, crude, and adjusted rates for the 9 parent-reported ACEs (Table 3). The total number of ACEs (range: 0–9) was summed to create an overall crude ACE score per individual and then reported by the percentage of children with 0, 1, ≥2, ≥3, ≥4, and ≥5 ACEs. Relatively small numbers of AI/AN necessitated using aggregate ACEs (e.g., ≥2, ≥3, ≥4, and ≥5) rather than single integrals of ACEs (e.g., 2, 3, 4, and 5).* Z* scores for the comparison of two proportions were used to determine the race-based differences in single and aggregate ACEs prevalence. Use of the NSCH ACE score has been established previously [15].

Second, we determined crude rate differences between AI/AN children with <2 ACEs compared to those with ≥2 ACEs and ≥3 ACEs as a function of selected health, developmental, and health service outcomes reported by the parent/caregiver (Table 4). The health, developmental, and service outcomes included those listed in Table 1. We calculated rate ratios and tested rate differences using the *Z* test for the comparison of two proportions.

Finally, logistic regression was performed to assess factors associated with the cumulative ACEs within the AI/AN population while controlling for possible confounders (Table 5). Two different models were developed to explore associations between child, family, and environmental characteristics and the following outcomes: (1) 2 or more ACEs and (2) parent-reported emotional, developmental, or behavioral conditions. The models produced adjusted odds ratios (AOR) for the sociodemographic characteristics (covariates) previously listed in Table 2. Multicollinearity diagnostics results did not identify problems with the inclusion of any of the above independent variables in the final models. The statistical analysis was conducted using SAS 9.3 survey procedures, which account for a complex sample design involving stratification, clustering, and multistage sampling.

## 3. Results

### 3.1. Sample

The sample characteristics are shown in Table 2 and indicate that, except for a limited number of variables (child's birth weight, gender, and CSHCN status), the AI/AN subsample was different in many ways compared to the NHW subsample. The AI/AN population had a higher percentage of children born ≥3 weeks prematurely (15.7% versus 11.0%) and a lower percentage of 12–17-year-olds (31.1% versus 36.6%). At the family level, the AI/AN population had a 3-fold higher proportion of incomes below 100% FPL (38.8% versus 11.3%) and a 3-fold lower proportion of incomes 400+% FPL (11.8% versus 36.5%). The AI/AN population was characterized by approximately 2.5 times higher proportion of households without a high school diploma (23.5% versus 9.2%) and a higher percentage of mothers <30 years of age (39.3% versus 22.9%). The household structure was more frequently reported to be a single mother with no father present (22.6% versus 12.5%) or some other arrangement such as living with grandparents, other close relatives, or foster parents (15.0% versus 5.9%). The child's health care coverage was more frequently public (57.6% versus 23.5%) or nonexistent (9.5% versus 3.9%) and the child was less likely to have a medical home (43.5% versus 65.7%). AI/AN families were more concentrated in nonmetropolitan statistical areas (40.3% versus 20.1%) and more likely to live in Alaska, the Northern Plains, and the Southwest compared to their NHW counterparts.

### 3.2. Prevalence of Adverse Childhood Experiences

Table 3 displays the individual and accumulated ACEs among AI/AN children and the comparison group of NHW children. Having been treated or judged unfairly based on race/ethnicity was approximately 7 times more common among AI/AN children than NHW children (10% versus 1.4%). AI/AN children were 2-3 times more likely to have a parent who served time in jail (18% versus 6%), to have observed domestic violence (15.5% versus 6.3%), to have been a victim of violence/witnessed violence in their neighborhood (15.9% versus 6.7%), and to have lived with a substance abuser (23.6% versus 11.6%). Finally, AI/AN children were 1.5 times more likely to live in families with difficulty covering basics like food or housing (35.7% versus 22.8%), to have lived with a divorced/separated parent (33% versus 21.4%), and to have lived with a parent who died (4.2% versus 2.5%). AI/AN and NHW children were equally likely to have lived with a mentally ill/suicidal/severely depressed person for more than a couple of weeks (13.2% versus 9.7%). Differences in accumulated ACEs were also evident. AI/AN children were less likely to have none of the 9 adverse experiences queried (35.2% versus 55.7%) and equally likely to have had one experience (24.5% versus 23.3%) but 2-3 times more likely to have multiple (≥2 to ≥5) ACEs (9.9 to 40.3% versus 3.3 to 21.0%) compared to NHW children.

To control for the many sociodemographic differences (confounders) between the AI/AN and NHW children (Table 2), the prevalence rates for the individual and accumulated ACEs were adjusted for all sociodemographic variables. Adjusted rates are presented in Table 3 and indicate that all statistically significant differences between the two populations are eliminated after adjustment.

### 3.3. Health/Developmental Problems and Services among AI/AN Children with Multiple ACEs

Table 4 displays the prevalence rates, prevalence rate differences, and prevalence rate ratios (PRR) for various health, developmental, and service outcomes among AI/AN children with <2 ACEs, 2+ ACEs, and 3+ ACEs. Among the parent-reported provider-diagnosed conditions, depression and anxiety disorders were significantly more prevalent among AI/AN children with 2+ ACEs (10.7% and 6.3%, resp.) compared with AI/AN children with <2 ACEs (0.4% and 1.8%, resp.) while these same emotional problems plus ADHD were more prevalent among AI/AN children with 3+ ACEs (14.4%, 7.7%, and 12.5%, resp.) compared to AI/AN children experiencing fewer than 2 ACEs (0.4%, 1.8%, and 5.5%, resp.). Prevalence rate ratios (PRR) for these same comparisons were the highest for depression (PRR = 26.8–36.0) and the lowest for ADHD (PRR = 2.0–2.3). AI/AN children with <2 ACEs were identified as having special health care needs 2.4 times less frequently than children with 2+ ACEs (13.6% versus 33.3%) and 2.8 times less often than children with 3+ ACEs (13.6% versus 38.0%). The likelihood of using prescribed medications, having elevated service needs, and having functional limitations significantly increased by 2.2–3 times for children with 2+ ACEs (23.4%, 14.4%, and 5.7%, resp.) and 2.1–3.6 times for children with 3+ ACEs (27.2%, 17.1%, and 5.4%, resp.) compared to those with <2 ACEs. In contrast, the likelihood of having a parent-reported emotional, behavioral, and developmental problem was 11.2 times greater for children with 2+ ACEs (19.1%) and 15.4 times for children with 3+ ACEs (26.1%) compared to children with <2 ACEs (1.7%).

Differences in parent-reported behavioral concerns among 0–5-year-old AI/AN children with <2 ACEs were generally similar to the young children with 2+ and 3+ ACEs. However, among AI/AN 6–17-year-olds, several parent-reported behavioral concerns showed increases in children with 2+ and 3+ ACEs compared to those with <2 ACEs: having problems in school (PRR = 2.1 and 2.0, resp.), arguing too much (PRR = 2.7 and 3.0, resp.), difficulty maintaining control in the face of challenges (PRR = 2.7 and 2.9, resp.), not caring about school performance (PRR = 2.8 and 2.8, resp.), and repeating grades (PRR = 2.5 and 2.9, resp.).

Regarding health care and service needs, receiving needed treatment or counseling was associated with higher likelihood values with accumulated numbers of ACEs from less than 2 to 2+ and 3+ ACEs. Children with 2+ (68.0%) and 3+ (66.7%) ACEs were approximately 3.5 times more likely to have received needed counseling than children with less than 2 ACEs (19.6%).

### 3.4. Associations with Multiple ACEs

The results of logistic regression to determine the relationship between individual, family, neighborhood, and residency factors and having 2+ ACEs are shown in Table 5. Adjusted odds ratios (AOR) indicating positive associations included age (AOR = 4.57 for 6–11-year-olds and AOR = 8.15 for 12–17-year-olds), family structure (AOR = 4.01 for single mothers, no father present; AOR = 4.85 for other family structures), public insurance (AOR = 2.23), neighborhood support (AOR = 1.74 for unsupportive neighborhoods), and region of the country (AOR = 2.18 for the Northern Plains states).

The results of an additional logistic regression analysis to determine the relationship between having emotional/developmental/behavioral problems as a function of individual, family, neighborhood, and residency factors and having 2+ ACEs are shown in Table 5. Results indicated that AI/AN children with 2+ ACEs had approximately 10 times greater odds of having parent-reported emotional/developmental/behavioral problems than AI/AN children with <2 ACEs. Independent effects were also noted for birth weight (AOR = 5.41 for low/very low birth weight) and family structure (AOR = 0.34 for single mother, no father present).

## 4. Discussion

The current analyses provide support, at least in part, for our hypotheses. First, we hypothesized that Native American children across the 0–17-year age range would be more likely to have greater accumulation of adverse experiences in childhood when compared to a reference population of NHW children in the same age range. Our results indicated that AI/AN children were more likely to have had 8 of 9 ACEs: income deprivation, witnessing or experiencing violent victimization, racial/ethnic discrimination, household substance abuse, domestic violence, parental incarceration, divorce, and death of a parent. Five of the 9 ACEs involved 2- to 7-fold crude rate increases in likelihood compared to the NHW population. AI/AN children were more likely to have multiple ACEs (≥2, ≥3, ≥4, and ≥5) when compared to non-Hispanic White children. However, after adjusting for sociodemographic factors, rate differences were eliminated, suggesting that elevated risk among the AI/AN child population was substantially accounted for by a combination of child, family, neighborhood, and residency factors used to adjust the models.

The results of this investigation are difficult to compare to other studies due to methodological and procedural differences. A limited number of ACE studies have been conducted with the AI/AN population and those that have are often focused on nonrepresentative samples of mature adults or larger studies of adolescents reporting on their own, possibly distant, past experiences [10–13]. Furthermore, in most studies of AI/AN adolescents and older AI/AN populations, the presence of ACEs is related to long-term outcomes such as alcohol/drug addiction, suicide attempts, intimate partner violence, and incarceration in a previously ACE-exposed population, thus elucidating the cyclic nature of these experiences [10–12, 16–18]. In addition, these adverse experience studies typically reference a broader array of traumatic events than what is covered in the current survey (e.g., sexual and psychological abuse, as well as various forms of neglect and boarding school attendance).

Due to this and other methodological factors, the results reported here are somewhat at odds with findings of race-based differences in ACEs revealed by other studies with adolescents and older AI/AN populations. For example, heightened risk of multiple victimization was associated with Native American males in a nationally representative sample of adolescents [13]. In that study, AI/AN children 12 years of age or older in Indian Country experienced 2-3 times the victimization rate of Whites, Blacks, and Asians. Two-thirds of the victimization cases were across racial boundaries, indicating a considerable amount of racial discrimination. Finally, rereferral rates to child protective services for abuse and neglect varied by racial and ethnic status with AI/AN families having the highest rates [19]. While some of these studies may have controlled for a variety of factors, none included the same extensive list of sociodemographic variables included here. As indicated by the differences outlined in Tables 2 and 3, the social and economic disparities between the AI/AN and the NHW children may contribute heavily to the crude rate differences in adverse events observed for the two racial/ethnic subgroups of children.

Our second hypothesis stated that the increasing accumulation of adverse events among AI/AN children would be associated with a gradient of health problems and need for services in the AI/AN population. This hypothesis was supported to some extent by our findings that accompanying the higher accumulation of adverse experiences among the AI/AN children was increasing prevalence of such problems reported by parents, particularly among children 6–17 years of age (e.g., arguing, lack of emotional control, and school problems) and more frequent provider-diagnosed behavioral disorders (depression, anxiety, and ADHD). AI/AN children with 2+ and 3+ ACEs received more medication and services such as counseling than AI/AN children with <2 ACEs.

The associated health and behavioral outcomes described here are reminiscent of those described by investigators of adolescents and older individuals. Similar to this investigation, one non-AI/AN study showed that, among younger (18–44 years), middle aged (45–64 years), and older (65–89 years) adults, increased ACE scores were associated with increased prescription medication dispensing rates for the treatment of depression and anxiety [20]. A study of 7 AI/AN tribes indicated a dose-response relationship with accumulated ACEs among AI/AN men and women [16]. That is, the number of different types of ACEs progressively increased the odds of having a negative outcome, such as alcohol dependence. Accumulated ACEs have also been found to be associated with increasing odds of attempted suicide and acts of violence among AI/AN women [12]. The diagnosis of PTSD (posttraumatic stress disorder) among AI/AN adolescents is also related to the number of adverse experiences [11].

To overcome the trauma of ACE in AI/AN communities, it has been suggested that a continuum of prevention strategies addressing primary, secondary, and tertiary needs is strongly needed [11]. First, efforts must be made to prevent new ACE occurrences; secondly, where ACEs have already occurred, efforts must focus on the prevention of risky behaviors in response to those experiences. Finally, those who have already developed a health problem as a long-term consequence of ACEs will need help to change health risk behaviors in order to lower the potential for disease burden. Furthermore, prevention and treatment behavioral health efforts must be (1) addressed by individuals, families, and communities, (2) integrated into community health systems, and (3) founded on evidence-, culture-, and practice-based approaches [21].

A few recent approaches introduced into health and social systems in AI/AN communities reflect adherence to these principles. In some cases, this has been accomplished by adapting evidence-based programs to incorporate AI/AN cultural values. For example, the Indian Country Child Trauma Center developed AI/AN adaptation of the evidence-based treatment, trauma-focused cognitive-behavioral therapy. Honoring Children, Mending the Circle (HC-MC) guides the therapeutic process through blending of AI/AN traditional teachings with cognitive-behavioral methods [22]. In other cases, nonadapted treatment protocols have been shown experimentally to be equally effective for an AI/AN subpopulation as for the targeted populations as a whole [23]. Finally, some programs have been specifically formulated for implementation in the AI/AN community.

Family Spirit intervention is an evidence-based AI/AN teen mother tribal home visiting program designed to address behavioral health disparities among American Indians and evaluated by using measures of intervention fidelity and early childhood emotional/behavioral development [24]. The latter targets maternal health, child development, school readiness, and positive parenting practices which are areas of emphasis in the Patient Protection and Affordable Care Act, Section 2951, addressing maternal, infant, and early childhood home visiting programs [25]. It is anticipated that home visiting programs in particular will address some of the underlying early life contributors to poor health and developmental outcomes for children, as well as persistent inequalities in the health and well-being of children and families. As the findings of this study indicate, the social determinants, especially those operating at the neighborhood, community, and regional levels, should figure prominently into the design of interventions aimed at improving the health of children and families. For example, the heavy concentration of unemployed AI/AN families relative to White families (15% versus 4.6% or 3.3 American Indian-to-White ratio) measured during the first half of 2013 in the Northern Plains region may have had some bearing on the doubling of the odds for ACEs compared to the East.

The findings presented here are subject to several limitations. The cross-sectional nature of the data imposes limits on the ability to discern any causal relationship between ACEs and the associated behaviors included here. In addition, due to the remote location and lower than average telephone service among many AI/AN families, sampling bias may reduce the representative nature of the AI/AN child sample from which estimates were drawn. Additional weaknesses include reliance on parent report for assigning children to diagnostic categories and evaluating functionality. These requirements may be difficult for parents in general but also may be fundamentally different for AI/AN parents compared to non-AI/AN parents based on cultural differences in the perception of disability that may lead to underreporting.

## 5. Conclusion

We have shown that significantly more AI/AN children 0–17 years of age are subject to adverse childhood experiences at a rate considerably higher and with greater complexity than a reference population of non-Hispanic White children. Increases in disease burden accompany those higher rates of adverse childhood experiences. Risks for some emotional, developmental, and behavioral problems in AI/AN children were increased relative to the reference group, though these were determined to be accounted for by social and economic factors. AI/AN children are more likely to experience multiple adverse events as they develop and their health behaviors are being shaped. Greater attention to the social determinants of health at the family, neighborhood, community, and higher levels of contextual influences such as those operating at the state, regional, or national levels is needed to address the marked health disparities shown here.

## Figures and Tables

**Table 1:** Definitions of parent-reported problem areas: National Survey of Children's Health 2011-2012.

Indicator	Definition
Provider-diagnosed conditions	These indicators are defined for children aged 2–17 years except for learning disability which was defined for children aged 3–17 years. *Questions*: Has a doctor or other health care provider ever told you that [child's name] had [insert disorder]? Does the child currently have [insert disorder]? (responses: 0 = no, 1 = yes) (i) Learning disability (ii) Depression (iii) Anxiety problems (iv) Behavioral or conduct problems (v) Autism, Asperger's disorder, pervasive developmental disorder, or other autism spectrum disorders (vi) Attention deficit disorder or attention deficit hyperactivity disorder (vii) Any developmental delay (viii) Speech or other language problems

Other parent-reported health conditions (screening questions for all surveyed children)	These indicators are defined for children aged 0–17 years. *Questions*: (i) Does [child's name] currently need or use medicine prescribed by a doctor, other than vitamins? (0 = no, 1 = yes) (ii) Does [child's name] need or use more medical care, mental health, or educational services than is usual for most children of the same age? (iii) Is [child's name] limited or prevented in any way in [his/her] ability to do the things most children of the same age can do? (iv) Does [child's name] need or get special therapy, such as physical, occupational, or speech therapy? (v) Does [child's name] have any kind of emotional, developmental, or behavioral problem for which [he/she] needs treatment or counseling?

Parent-reported child behaviors (0–5 years)	These indicators are defined for children aged 0–5 years. *Questions*: Do you have any concerns about [child's name]'s learning, development, or behavior? (0 = a little/not at all, 1 = a lot) Are you concerned a lot, a little, or not at all about how [he/she] (i) Is learning to do things for [himself/herself]? (ages 10 months to 5 years) (ii) Is learning preschool or school skills? (ages 18 months–5 years) (iii) Behaves? (ages 4 months–5 years) (iv) Gets along with others? (ages 4 months–5 years)

Parent-reported child behaviors (6–17 years)	These indicators are defined for children aged 6–17 years. *Questions*: During the past 12 months, how many times has [child's name]'s school contacted you or another adult in your household about any problems [he/she] is having with school? (0 = 0 times, 1 = ≥1 time) The following is a list of items that sometimes describe children. For each item, please tell me how often this was true for [child's name] during the past month. Would you say never, rarely, sometimes, usually, or always true for [child's name] during the past month? [He/she] argues too much (0 = never/rarely/sometimes, 1 = usually/always) [He/she] bullies or is cruel or mean to others (0 = never/rarely/sometimes, 1 = usually/always) [He/she] is unhappy, sad, or depressed (0 = never/rarely/sometimes, 1 = usually/always) [He/she] stays calm and in control when faced with a challenge (1 = never/rarely/sometimes, 0 = usually/always) [He/she] cares about doing well in school (1 = never/rarely/sometimes, 0 = usually/always) Since starting kindergarten, has [he/she] repeated any grades? (0 = no, 1 = yes)

Health care services	These indicators are defined for children aged 0–17 years. *Questions (adequate insurance = usually/always for each of the 3 questions)*: Does [child's name]'s health insurance offer benefits or cover services that meet [his/her] needs? (0 = never/rarely/sometimes, 1 = usually/always) Does [child's name]'s health insurance allow [him/her] to see the health care providers [he/she] needs? (0 = never/rarely/sometimes, 1 = usually/always) How often are these costs reasonable? (0 = never/rarely/sometimes, 1 = usually/always) *Questions (received needed counseling = yes to both questions)*: Does [child's name] have any kind of emotional, developmental, or behavioral problem for which [he/she] needs treatment or counseling? (0 = no, 1 = yes) Mental health professionals include psychiatrists, psychologists, psychiatric nurses, and clinical social workers. During the past 12 months, has [child's name] received any treatment or counseling from a mental health professional? (0 = no, 1 = yes) *Questions (developmental screening = yes to all questions)*: During the past 12 months, did a doctor or other health care providers have you fill out a questionnaire about specific concerns or observations you may have about [child's name]'s development, communication, or social behaviors? Did this questionnaire ask about your concerns or observations about How [child's name] talks or makes speech sounds? (0 = no, 1 = yes, ages 10–23 months) How [child's name] interacts with you and others? (0 = no, 1 = yes, ages 10–23 months) Words and phrases [child's name] uses and understands? (0 = no, 1 = yes, ages 24–71 months) How [child's name] behaves and gets along with you and others? (0 = no, 1 = yes, ages 24–71 months) *Questions (developmental concerns = yes to one question)*: [During the past 12 months/since [child's name]'s birth], did [child's name]'s doctors or other health care providers ask if you have concerns about [his/her] learning, development, or behavior? (0 = no, 1 = yes) *Questions (child has IFSP or IEP = yes to either question)*: Does [child's name] have any developmental problems for which [he/she] has a written intervention plan called an [if age < 36 months, insert: Individualized Family Services Plan or an IFSP?; if age ≥ 36 months, insert: Individualized Education Program or IEP?] Does [child's name] have a health problem, condition, or disability for which [he/she] has a written intervention plan called an Individualized Education Program or IEP?

**Table 2:** Sample characteristics of American Indian/Alaska Native and non-Hispanic White children 0–17 years of age: National Survey of Children's Health 2011-2012.

Characteristic	American Indian/Alaska Native				Non-Hispanic White				*P*-value for prevalence rate difference^*∗*^
	Unweighted *N*	Weighted *N*	Prevalence		Unweighted *N*	Weighted *N*	Prevalence		
			%	Standard error			%	Standard error	
Total	1,453	686,615			61,381	37,666,681			

Child's birth weight									
Within normal limits	1,230	582,057	91.1	1.81	54,802	33,628,030	92.5	0.21	0.441
Low	97	49,637	7.8	1.79	3,842	2,361,119	6.5	0.20	0.472
Very low	27	7,176	1.1	0.32	680	356,910	1.0	0.08	0.764

Child's gestation									
Within normal limits	1,229	575,906	84.3	2.07	54,511	33,390,165	89.0	0.26	0.024
3 or more weeks' premature	206	107,150	15.7	2.07	6,592	4,122,349	11.0	0.26	0.024

Child's gender									
Male	743	371,833	54.2	2.82	31,651	19,386,409	51.6	0.42	0.36
Female	709	314,306	45.8	2.82	29,660	18,215,698	48.4	0.42	0.36

Child's age									
0–5 years	499	226,098	32.9	2.51	18,228	11,742,994	31.2	0.39	0.503
6–11 years	480	246,799	35.9	2.92	19,455	12,148,511	32.3	0.39	0.223
12–17 years	474	213,719	31.1	2.70	23,698	13,775,176	36.6	0.41	0.044

CSHCN status									
CSHCN	325	147,732	21.5	2.48	13,079	8,130,399	21.6	0.35	0.968
Non-CSHCN	1,128	538,883	78.5	2.48	48,302	29,536,282	78.4	0.35	0.968

Household income (FPL)^†^									
<100%	548	266,374	38.8	2.97	5,566	4,267,420	11.3	0.28	>0.0001
100–199%	371	203,958	29.7	2.99	9,558	7,034,578	18.7	0.35	0.0003
200–399%	335	135,545	19.7	2.09	20,532	12,633,812	33.5	0.41	>0.0001
400+%	200	80,738	11.8	1.68	25,724	13,730,871	36.5	0.40	>0.0001

Family structure									
Two parents, biological/adopted	735	344,177	50.6	2.91	46,578	27,202,154	72.5	0.40	>0.0001
Two parents, step family	149	80,469	11.8	1.64	4,111	3,424,912	9.1	0.27	0.105
Single mother, no father present	309	153,849	22.6	2.36	6,892	4,671,895	12.5	0.28	>0.0001
Other	247	102,095	15.0	2.36	3,550	2,222,396	5.9	0.22	0.0001

Mother's age									
30 years or less	587	270,009	39.3	2.81	11,943	8,621,552	22.9	0.37	>0.0001
31–45 years	615	352,373	51.3	2.88	33,595	21,686,721	57.6	0.42	0.031
>45 years	251	64,233	9.4	1.21	15,843	7,358,407	19.5	0.31	>0.0001

Highest household education level									
Less than high school	284	147,493	23.5	2.78	4,926	3,355,530	9.2	0.26	>0.0001
High school	501	195,741	31.2	2.44	21,374	12,983,089	35.5	0.40	0.082
More than high school	543	284,028	45.3	3.11	33,522	20,256,886	55.4	0.42	0.001

Child's insurance coverage									
Public	814	385,801	57.6	2.91	12,437	8,755,041	23.5	0.37	>0.0001
Private	480	220,824	32.9	2.80	46,452	27,088,341	72.6	0.40	>0.0001
No coverage	124	63,601	9.5	1.58	1,999	1,463,918	3.9	0.19	0.0004

Medical home									
Has a medical home	603	292,428	43.5	2.92	40,374	24,137,715	65.7	0.41	>0.0001
Does not have a medical home	808	379,495	56.5	2.92	19,433	12,581,727	34.3	0.41	>0.0001

Neighborhoods									
Supportive	1,146	530,325	77.5	2.15	54,832	33,042,224	88.0	0.28	>0.0001
Nonsupportive	296	153,605	22.5	2.15	6,283	4,490,565	12.0	0.28	>0.0001

Metropolitan status									
Within a MSA	605	401,320	59.7	2.65	43,437	29,818,692	79.9	0.30	>0.0001
Not within a MSA	836	271,470	40.3	2.65	17,545	7,522,853	20.1	0.30	>0.0001

AI/AN regions									
Alaska	261	28,292	4.1	0.41	1,021	99,662	0.3	0.01	>0.0001
East	457	318,387	46.4	2.95	37,310	26,108,226	69.3	0.34	>0.0001
Northern Plains	486	132,977	19.4	1.85	12,799	4,769,166	12.7	0.17	0.0003
Pacific Coast	70	56,799	8.3	1.50	4,993	4,327,345	11.5	0.33	0.0380
Southwest	179	150,160	21.9	2.14	5,258	2,362,282	6.3	0.11	>0.0001

^*∗*^Probability for comparison of American Indian/Alaska Native compared to non-Hispanic White children based on the *Z* test for the comparison of two proportions.

^†^Federal poverty levels (FPL) are based on the Department of Health and Human Services 2012 poverty guidelines. Income below 100% of the poverty threshold was defined as less than $15,130 for a family of two, $19,090 for a family of three, and $23,050 for a family of four.

**Table 3:** Unadjusted (crude) and adjusted rates of parent-reported adverse childhood experiences (ACEs) among American Indian/Alaska Native children compared to non-Hispanic white children: National Survey of Children's Health 2011-2012.

Adverse childhood experiences	Non-Hispanic American Indian/Alaska Native	Non-Hispanic White	*P* value for unadjusted prevalence rate difference^*∗*^	Non-Hispanic American Indian/Alaska Native	Non-Hispanic White	*P* value for adjusted prevalence rate difference^*∗∗*^
	Unadjusted prevalence	Unadjusted prevalence		Adjusted prevalence	Adjusted prevalence	
	% (SE)	% (SE)		% (SE)	% (SE)	
Child's family has difficulty getting by on the family's income, hard to cover basics like food or housing	35.7 (2.99)	22.8 (0.38)	>0.0001	18.3 (3.52)	21.9 (0.62)	0.313
Child lived with parent who got divorced/separated after he/she was born	33.0 (2.85)	21.4 (0.37)	0.0001	20.1 (3.01)	21.2 (0.57)	0.719
Child lived with anyone who had a problem with alcohol or drugs	23.6 (2.65)	11.6 (0.29)	>0.0001	8.1 (2.33)	10.0 (0.46)	0.424
Child lived with parent who served time in jail after he/she was born	18.0 (2.60)	6.0 (0.21)	>0.0001	7.5 (3.64)	5.4 (0.37)	0.569
Child was a victim of violence or witnessed violence in his/her neighborhood	15.9 (2.39)	6.7 (0.21)	0.0001	9.2 (2.69)	5.4 (0.35)	0.162
Child saw parents hit, kick, slap, punch, or beat each other up	15.5 (2.58)	6.3 (0.22)	0.0004	8.6 (2.43)	5.7 (0.38)	0.238
Child lived with anyone who was mentally ill or suicidal or severely depressed for more than a couple of weeks	13.2 (2.26)	9.7 (0.26)	0.124	5.7 (2.10)	8.7 (0.45)	0.162
Child was ever treated or judged unfairly because of his/her race or ethnic group	10.0 (1.45)	1.4 (0.10)	>0.0001	5.2 (2.07)	0.9 (0.14)	0.039
Child lived with parent who died	4.2 (0.73)	2.5 (0.14)	0.022	1.4 (0.59)	2.2 (0.19)	0.197
Number of ACEs among participating children, of 9 asked about						
0	35.2 (2.66)	55.7 (0.43)	>0.0001	56.2 (5.11)	58.1 (0.64)	0.711
1	24.5 (2.60)	23.3 (0.38)	0.646	43.8 (5.11)	41.9 (0.64)	0.711
≥2	40.3 (2.87)	21.0 (0.36)	>0.0001	22.0 (4.11)	19.0 (0.57)	0.472
≥3	26.8 (2.72)	11.5 (0.29)	>0.0001	12.9 (2.37)	10.3 (0.48)	0.280
≥4	16.8 (2.55)	6.2 (0.22)	>0.0001	3.6 (1.30)	5.4 (0.38)	0.184
≥5	9.9 (2.18)	3.3 (0.16)	0.003	2.3 (0.97)	2.7 (0.28)	0.689

^*∗*^Probability for comparison of American Indian/Alaska Native compared to non-Hispanic White children unadjusted (crude) ACE prevalence rates based on the *Z* test for the comparison of two proportions.

^*∗∗*^Probability for comparison of American Indian/Alaska Native compared to non-Hispanic White children adjusted ACE rates based on the *Z* test for the comparison of two proportions. Rates were adjusted for birth weight, prematurity, sex, age, special needs status, poverty, family structure, mother's age, highest household educational level, type of insurance coverage, medical home access, neighborhood support, region, and metropolitan status.

**Table 4:** Developmental, behavioral, and service-related outcomes of AI/AN children with fewer than 2 parent-reported ACEs compared to AI/AN children with 2 or more and 3 or more parent-reported ACEs: National Survey of Children's Health 2011-2012.

Developmental, behavioral, and service-related outcomes	Less than 2 ACEs among AI/AN children	2 or more ACEs among AI/AN children	*P* value for prevalence rate difference^*∗*^	Prevalence rate ratio^*∗*^	3 or more ACEs among AI/AN children	*P* value for prevalence rate difference^*∗∗*^	Prevalence rate ratio^*∗∗*^
	%	%			%		
Provider-diagnosed conditions							
Learning disability	9.9 (3.21)	11.7 (2.42)	0.653	1.2	13.1 (3.22)	0.484	1.3
Depression	0.4 (0.35)	10.7 (4.82)	0.033	26.8	14.4 (6.87)	0.041	36.0
Anxiety disorder	1.8 (0.97)	6.3 (1.81)	0.029	3.5	7.7 (2.53)	0.029	4.3
Conduct disorder	1.4 (0.56)	3.6 (1.09)	0.072	2.6	4.6 (1.56)	0.054	3.3
Autism spectrum disorder	1.1 (0.44)	1.3 (0.43)	0.741	1.2	1.5 (0.56)	0.576	1.4
ADHD	5.5 (1.83)	10.8 (2.16)	0.062	2.0	12.5 (2.93)	0.042	2.3
Developmental delay	3.8 (0.96)	3.5 (0.81)	0.810	0.9	4.0 (1.07)	0.889	1.1
Speech disorder	5.0 (1.08)	7.3 (2.01)	0.313	1.5	7.4 (2.56)	0.390	1.5

Other parent-reported health conditions/needs							
Special health care needs	13.6 (2.24)	33.3 (4.72)	0.0002	2.4	38.0 (6.35)	0.0003	2.8
Prescribed medication	9.2 (1.95)	23.4 (4.70)	0.005	2.5	27.2 (6.51)	0.008	3.0
Elevated service use	4.8 (1.11)	14.4 (2.59)	0.0006	3.0	17.1 (3.60)	0.001	3.6
Functional limitations	2.6 (0.71)	5.7 (1.25)	0.031	2.2	5.4 (1.41)	0.077	2.1
Special therapies	3.2 (0.80)	5.0 (1.52)	0.294	1.6	5.9 (2.19)	0.246	1.8
Emotional, developmental, or behavioral problems	1.7 (0.40)	19.1 (4.78)	0.0003	11.2	26.1 (6.64)	0.0002	15.4

Parent-reported child behaviors (0 to 5 years of age)							
Parent is concerned a lot about the following							
How the child is learning to do things for him-/herself	4.8 (1.63)	2.3 (1.20)	0.215	0.5	5.0 (2.69)	0.952	1.0
How the child is learning in preschool	5.6 (1.95)	3.3 (1.41)	0.337	0.6	7.3 (3.19)	0.653	1.3
How the child behaves	8.5 (3.44)	6.5 (3.02)	0.66	0.8	7.8 (3.14)	0.881	0.9
How the child gets along with others	9.7 (3.48)	2.1 (1.07)	0.037	0.2	4.9 (2.53)	0.263	0.5

Parent-reported child behaviors (6 to 17 years of age)							
Child has been having problems with school	22.5 (3.41)	48.2 (5.45)	0.0001	2.1	45.4 (6.72)	0.002	2.0
Child argues too much	11.4 (2.23)	30.3 (5.37)	0.001	2.7	34.5 (6.98)	0.002	3.0
Child bullies or is cruel or mean to others	2.0 (1.11)	4.5 (2.29)	0.327	2.3	6.0 (3.20)	0.238	3.0
Child is unhappy, sad, or depressed	2.7 (1.41)	5.6 (3.46)	0.435	2.1	6.3 (4.73)	0.465	2.3
Child does not maintain self-control when faced with a challenge	3.6 (1.13)	9.7 (2.42)	0.023	2.7	10.5 (3.12)	0.038	2.9
Child does not care about doing well in school	11.1 (2.01)	31.0 (4.31)	>0.0001	2.8	31.6 (5.39)	0.0004	2.8
Child repeated grade	9.0 (2.27)	22.3 (5.52)	0.026	2.5	26.2 (7.28)	0.024	2.9

Health care/services							
Child has insurance adequate to cover needed services	78.6 (3.88)	76.5 (3.68)	0.697	1.0	75.3 (5.01)	0.603	1.0
Child received needed counseling	19.6 (6.55)	68.0 (9.69)	>0.0001	3.5	66.7 (10.8)	0.0002	3.4
Child was screened by a doctor for developmental problems	22.9 (4.13)	30.5 (9.43)	0.459	1.3	24.8 (10.0)	0.857	1.1
Doctor asked about parent concerns	50.2 (5.00)	52.9 (8.63)	0.787	1.1	46.5 (10.4)	0.749	0.9
Child has IEP or IFSP	9.2 (1.60)	13.1 (2.43)	0.18	1.4	13.1 (3.01)	0.254	1.4

^*∗*^Probability for comparison of 2+ ACEs versus fewer than 2 ACEs based on the *Z* test for the comparison of two proportions. ^*∗∗*^Probability for comparison of 3+ ACEs versus fewer than 2 ACEs based on the *Z* test for the comparison of two proportions.

**Table 5:** Adjusted odds ratios for sociodemographic characteristics associated with (1) having 2 or more ACEs and (2) having parent-reported emotional/developmental/behavioral problems among AI/AN children 0–17 years of age: National Survey of Children's Health 2011-2012.

Sociodemographic characteristics	Outcome: two or more adverse family experiences		Outcome: parent-reported emotional/developmental/behavioral problems	
	Adjusted odds ratio	95% confidence limits	Adjusted odds ratio	95% confidence limits
Adverse childhood events				
2 or more ACEs	na	na	**10.3**	3.64–29.3
Less than 2 ACEs	na	na	1.00	Reference

Birth weight				
Within normal limits	1.00	Reference	1.00	Reference
Low/very low birth weight	0.72	0.23–2.25	**5.41**	1.48–19.8

Child's gestation				
Within normal limits	1.56	0.60–4.05	1.00	Reference
3 or more weeks' premature	1.00	Reference	1.61	0.48–5.37

Child's gender				
Male	0.84	0.50–1.41	1.30	0.60–2.81
Female	1.00	Reference	1.00	Reference

Child's age				
0–5 years	1.00	Reference	1.00	Reference
6–11 years	**4.57**	2.23–9.36	1.04	0.42–2.60
12–17 years	**8.15**	3.55–18.7	1.89	0.66–5.43

Household income (FPL)^†^				
<100%	1.51	0.51–4.47	1.22	0.33–4.51
100–199%	1.31	0.43–3.94	1.71	0.49–5.94
200–399%	1.09	0.42–2.80	0.66	0.18–2.40
400+%	1.00	Reference	1.00	Reference

Family structure				
Two parents, biological/adopted	1.00	Reference	1.00	Reference
Two parents, step family	2.04	0.97–4.32	1.05	0.38–2.90
Single mother, no father present	**4.01**	2.00–8.03	**0.34**	0.12–0.94
Other	**4.85**	1.99–11.8	0.63	0.18–2.16

Mother's age				
30 years or less	1.06	0.41–2.70	0.86	0.21–3.53
31–45 years	1.27	0.63–2.56	1.30	0.46–3.65
>45 years	1.00	Reference	1.00	Reference

Highest household education level				
Less than high school	0.73	0.37–1.47	0.88	0.38–2.90
High school	1.03	0.61–1.78	0.88	0.39–1.96
More than high school	1.00	Reference	1.00	Reference

Child's insurance coverage				
Public	**2.23**	1.17–4.26	1.13	0.43–2.95
No coverage	1.89	0.64–5.59	0.91	0.21–3.95
Private	1.00	Reference	1.00	Reference

Medical home				
Has a medical home	1.00	Reference	1.00	Reference
Does not have a medical home	1.17	0.70–1.94	2.03	0.86–4.79

Neighborhoods				
Supportive	1.00	Reference	1.00	Reference
Nonsupportive	**1.74**	1.01–3.00	0.70	0.29–1.68

Metropolitan status				
Within a MSA	1.00	Reference	1.00	Reference
Not within a MSA	0.92	0.56–1.52	0.58	0.26–1.32

AI/AN regions				
Alaska	1.06	0.56–2.00	1.69	0.68–4.21
East	1.00	Reference	1.00	Reference
Northern Plains	**2.18**	1.21–3.94	0.76	0.31–1.85
Pacific Coast	1.49	0.58–3.85	1.95	0.51–7.53
Southwest	1.26	0.63–2.53	0.69	0.24–1.98

Wald *χ* ^2^ statistic and *P* value	96.5	<0.0001	46.6	0.005
*R* ^2^ (Nagelkerke max-rescaled *R*-square)	0.85		0.49	

^†^Federal poverty levels (FPL) are based on the Department of Health and Human Services 2012 poverty guidelines. Income below 100% of the poverty threshold was defined as less than $15,130 for a family of two, $19,090 for a family of three, and $23,050 for a family of four.
